# Interpreting whole-genome sequencing data during neonatal *Klebsiella oxytoca* complex outbreak management

**DOI:** 10.1186/s13756-025-01595-6

**Published:** 2025-07-01

**Authors:** Chiara Minotti, Elena Robinson, Pascal Schlaepfer, Christian Pohl, Daniel Goldenberger, Sven M. Schulzke, Peter Michael Keller, Julia Anna Bielicki

**Affiliations:** 1 Infectious Disease and Vaccinology Unit and Paediatric Research Centre, University Children’s Hospital Basel UKBB, Spitalstrasse 33, Basel, 4031 Switzerland; 2 Department of Clinical Research DKF, Faculty of Medicine, University of Basel, Basel, Switzerland; 3 University of Basel, Basel, Switzerland; 4 University of Zurich, Zurich, Switzerland; 5 Laboratory Medicine, University Hospital Basel, Basel, Switzerland; 6 Department of Neonatology, University Children’s Hospital Basel UKBB, Basel, Switzerland; 7 Clinical Bacteriology and Mycology, University Hospital Basel, Basel, Switzerland; 8 Centre for Neonatal and Paediatric Infection, Institute for Infection & Immunity, City St. George’s University of London, London, UK

**Keywords:** Outbreak containment, WGS, Genotyping, *Klebsiella oxytoca*, Newborn, Environmental source

## Abstract

**Background:**

*K. oxytoca* generally has a benign susceptibility profile and low virulence but can cause invasive infections in vulnerable populations, like preterm infants. We aim to describe how whole-genome sequencing (WGS) was used to inform management of a prolonged *K. oxytoca* outbreak on a neonatal intensive care unit (NICU) and implications for outbreak response involving similar organisms.

**Methods:**

We retrospectively reviewed outbreak-associated clinical and environmental isolates from a Swiss NICU. WGS was used to track evolution of resistance and highlighted multiple concurrent outbreaks. WGS was performed using a MiSeq or NextSeq 500 Illumina sequencer. The resulting genome sequences were analysed using Ridom SeqSphere. The current report conforms to ORION reporting guidelines.

**Results:**

Of 152 *Klebsiella* spp. patient-derived isolates, 83 were genotyped using WGS, along with six environmental isolates. This confirmed two outbreak waves (November 2021-February 2022, ST18 wildtype; July 2022-June 2023, main cluster ST18 KI β-lactamase hyperproducer), with multiple genotypically connected clusters during the second wave. Confirmed sepsis (*K. oxytoca* ST18 wildtype) occurred in four preterm or low birthweight infants. Twins presented a genotypically identical ST with a different susceptibility phenotype (ST18 wildtype vs. K1 OXY-hyperproducer). WGS combined with epidemiological investigation and environmental sampling identified an environmental source. There was a second outbreak wave after source removal, presumably due to the prolonged presence of colonised infants with typically long NICU stays and insufficient standard infection prevention and control measures to prevent transmission.

**Conclusion:**

WGS use in NICU outbreaks involving low-virulence bacteria can support identification and removal of potentiating environmental sources. These measures, however, will often be insufficient to contain the outbreak, and ongoing WGS surveillance of ubiquitous species may uncover multiple concurrent outbreaks, presumably driven by continuing transfer-transmission between different sources and infants in the NICU. Maximising standard infection prevention and control (IPC) measures is appropriate in this context.

**Supplementary Information:**

The online version contains supplementary material available at 10.1186/s13756-025-01595-6.

## Background

Neonatal unit outbreaks caused by Enterobacterales and especially by *Klebsiella* spp. are increasingly reported [1–3]. *K. oxytoca* complex includes at least six species (*K. grimontii*, *K. huaxiensis*, *K. michiganensis*, *K. oxytoca*, *K. pasteurii* and *K. spallanzanii*). All species are human commensals, notably ubiquitous, and potential opportunistic pathogens, described as being responsible for nosocomial outbreaks [2, 4]. Possible sources and nosocomial reservoirs have been described, and it has also been shown that *K. oxytoca* can survive transiently on hands [1, 2].

*K. oxytoca* generally has a favourable susceptibility profile and low virulence but can cause invasive infections in vulnerable populations, like preterm newborns and infants admitted to neonatal intensive care units (NICUs) [5–8]. Despite being recognised as a pathogen in susceptible, high-risk populations in healthcare settings, the lack of comprehensive surveillance data on *K. oxytoca* makes it challenging to accurately estimate the incidence of neonatal outbreaks across Europe and globally [2, 8]. A recent outbreak report including a systematic review has identified nine further NICU outbreak reports involving *K. oxytoca*, mostly located in Europe [3]*.* Cases of neonatal invasive infection have been described along colonisation and identification in environmental sources, reporting antimicrobial resistance in the majority of the available reports, generally with a long time-to-resolution (from two months to over one year) (Table 1).

**Table 1:** Research in context

**Evidence before this study**
*K. oxytoca* is a low-virulence pathogen, reported as a cause of invasive hospital-acquired infections in vulnerable populations, such as preterm infants. There is a lack of comprehensive surveillance data on *K. oxytoca*, making it challenging to accurately estimate the incidence of neonatal outbreaks across Europe and globally. We searched Ovid Embase, Ovid MEDLINE and Scopus, without date or language restrictions (last search on 23.01.2025), with search terms [neonat* OR newborn*] AND [infection* OR sepsis OR septic* OR “nosocomial infection”] along with [center* OR unit(s) OR nursery OR nurseries OR hospital* OR NICU OR neonatology] AND [outbreak* OR epidemic* OR cluster*] AND ["Klebsiella oxytoca"OR"K. oxytoca”]. The full search strategy is available in the Supplementary Materials. We further performed a manual search of reference lists for relevant publications. We included *K. oxytoca* complex outbreak reports in neonatal intensive care units (NICUs), neonatal units or neonatal special care units*.* Nearly two-thirds of twenty relevant reports were from Europe. Antibiotic resistance was commonly described, with most recent studies reporting a majority of extended-spectrum-β-lactamase (ESBL)-producing strains, followed by K1 [hOXY] phenotype and Verona integron-mediated metallo-β-lactamase (VIM carbapenemase) producing. Just over half of the reports described colonisation rates over 50%, and most described at least one case of infection, especially bacteraemia. Probable outbreak sources were identified in a minority of studies. Several different genotyping and sequencing techniques were described for outbreak characterisation from 1994 onwards, often with multiple techniques per study with whole genome sequencing uncommon. Identified STs were ST179, ST201, ST11, ST308, ST389, ST392 (*K. oxytoca*), and ST50 (*K. michiganensis*)
**Added value of this study**
We describe how whole-genome sequencing (WGS) was used to inform management of a biphasic *K. oxytoca* outbreak in a Swiss NICU. WGS revealed or refuted epidemiological links, highlighted multiple concurrent outbreaks with an otherwise low-virulence pathogen and enabled tracking of evolution of resistance. The identification of potential environmental sources is often difficult and not frequently described in published reports. In the NICU, likely multidirectional transfer-transmission between environmental sources and infants means that source identification and removal may not be sufficient to contain an outbreak*.* Indeed, after removal of the identified environmental source there was persisting potential for onward infant-to-infant transmission due to typically long NICU stays and consequent prolonged presence of colonised infants, leading to a second outbreak wave. Furthermore, twins presented a genotypically identical ST with a different susceptibility phenotype, wildtype vs. K1. (hyperproducer of OXY [hOXY-KoC], following point mutations in the consensus sequences of their promoters). This pertains to the emergence of resistance during an outbreak in a pathogen that is otherwise well-treatable
**Implications of all the available evidence**
The use of WGS in the context of NICU outbreaks involving low-virulence bacteria can support identification and removal of potentiating environmental sources. These measures, however, will often be insufficient to fully contain the outbreak. Ongoing WGS surveillance of ubiquitous species may uncover multiple concurrent outbreaks, driven by ongoing transfer-transmission between different sources and infants. Cost-efficient outbreak response, especially for low-virulence pathogens, must combine outbreak-specific investigations with maximisation of standard measures, such as optimal hand hygiene practices. Hand hygiene with appropriate monitoring to boost compliance would significantly reduce multiple transfer-transmission events. Collaborations between infection prevention and control (IPC) and microbiology teams would enable the identification of tailored measures to address outbreak risks to patients and expected efficiency of IPC measures

Timely identification of outbreaks, their efficient management and targeted infection prevention and control (IPC) measures can minimise adverse patient impacts. Among tools used for outbreak characterisation and transmission patterns investigation, whole-genome sequencing (WGS), combined with epidemiological investigation, can reveal or refute epidemiological links, enable tracking of evolution of resistance and highlight multiple concurrent outbreaks.

We aim to describe how WGS was used to inform management of a biphasic *K. oxytoca* outbreak on a Swiss third-level NICU and implications for outbreak response involving similar organisms.

## Materials and methods

### Setting and outbreak detection

This is an outbreak report, reflecting measures implemented in real practice to contain the outbreak. The outbreak occurred in the 32-cot, level III NICU of the University Children’s Hospital Basel in Basel, Switzerland, offering routine care to extremely preterm infants (< 28 weeks’ gestational age) with ~ 600 neonates admitted yearly.

For the current report, medical and laboratory records were retrospectively reviewed and relevant clinical data extracted systematically. An outbreak was defined as the occurrence of at least two epidemiologically and genotypically related clinical cases in the context of continuous genomic evaluation of invasive isolates in our institution. Infants were considered confirmed outbreak cases if molecular subtyping with WGS revealed a clear genotypic link between isolates, whether from clinical or screening cultures. Inpatients positive for any *K. oxytoca* strain in any specimen were considered potential outbreak cases. We further distinguished between infection cases (clinically symptomatic with positive cultures), and colonised infants, defined as the positivity of surveillance swabs (mostly rectal) without associated clinical symptoms.

Infants are routinely screened for multidrug-resistant organisms (MDRO) at 2–3 days and 4 weeks of life, if still hospitalised on the unit. During the first outbreak wave, an intensive and targeted screening was implemented to identify susceptible *K. oxytoca* strains, that would have been missed with baseline screening. Subsequently, during the second wave additional screening timepoints were introduced (at birth and one week of life), targeting susceptible *Klebsiella* spp. and non-susceptible, K1-producing *Klebsiella* strains. Infants were screened multiple times during the outbreak, thus contributing to longitudinal data. If presenting multiple positive samples in time, only the first positive sample was considered for this report. Routine water testing takes place twice a year, including tap filters and sinks outside the rooms. Targeted screening was implemented during the two outbreak waves (during the first wave to identify the source/potentiator, then in the second wave to confirm no further source had been overlooked). Agents used for disinfection were alcohol-based hand rubs (Skinman™/Sterilium®pure) for hand sanitising; alcohol-based tissues (Bacillol® AF Tissues) and solutions (Incidin™ Pro, bactericidal, virucidal) for surface disinfection. The nurse-to-patient ratio is 1:3 to 1:2/1:1 (the latter in case of critical patients).

WGS is routinely applied during outbreak investigations. Eighty-three out of 152 *Klebsiella* spp. isolates cultured from patient samples were genotyped using WGS, along with six environmental isolates. The sequencing results have been uploaded on the National Centre for Biotechnology Information (NCBI) under project number PRJNA1198653.

### Microbiological and molecular methods

Following clinical suspicion of invasive infection, blood samples were taken as part of routine sepsis work-up. As per routine practice, blood was cultured at 37 °C for up to five days in an aerobic BacT/ALERT culture medium (bioMérieux, France). Upon bacterial growth, a subculture was done. Rectal swabs were obtained as part of outbreak investigation to assess prevalence of colonisation by the outbreak strain. The samples were cultured for 48 h at 37 °C on a 5% sheep-blood agar plate. After colony growth and preliminary morphological assessment of *Klebsiella* spp., species identification was performed on 9–20 colonies per infant using MALDI-TOF MS with a MALDI Biotyper® sirius or microflex system (Bruker Daltonics, Bremen, Germany). Environmental samples, including components of bathtubs and water, were tested for Enterobacterales according to ISO 21528–2 and species identification in positive samples was conducted with MALDI-TOF–MS (Shimadzu, Japan). For antimicrobial susceptibility testing, VITEK2 (BioMérieux, France) was used. After identifying *K. oxytoca*, a pool of 3–5 colonies underwent WGS, which was performed using a MiSeq or NextSeq 500 Illumina sequencer. The resulting genome sequences were analysed using Ridom SeqSphere (v8 4.1). Both sequencing and data analysis are accredited according to the ISO/IEC 17025 standard.

DNA was extracted with the EZ1 Advanced XL (QIAGEN) and then sequenced on the Illumina NexSeq 500 platform. Raw reads were quality-checked with FastQC (v0.11.9) and trimmed with Trimmomatic (v0.36) to remove adaptors. Draft assemblies were generated with Unicycler (v0.4.8) following the Illumina-only assembly pipeline and quality checked with QUAST (v5.2.0).

In silico screening for antimicrobial resistance genes (ARGs) and replicon sequences was performed using AMRFinder, ResFinder v4.1, and PlasmidFinder v2.1 (50% minimum percentage identity) software from the Center for Genomic Epidemiology (CGE), and RIDOM Seqsphere + v8.5.1, respectively. Multilocus sequence typing (MLST) was done with MLST v2.0 (CGE) and the *K. oxytoca* species complex typing database (PubMLST) respectively with RIDOM Seqsphere +. Accurate species confirmation was conducted with the Type Strain Genome Server (TYGS). The *bla*_*OXY*_ genes were annotated according to the *Klebsiella* locus/sequence definitions database from the Institut Pasteur (BIGSdb-Pasteur). To characterise the promoter sequences, draft assemblies were annotated with Prokka (v1.13), and the contigs containing the *bla*_*OXY*_ were extracted with a custom perl script. The upstream regions (−33 to −32 bp) of the *bla*_*OXY*_ were manually scanned for the −35 (TTGTCA), 17 bp spacer, and −10 (GATAGT, GATAAT, TATAGT, and TATACT) promoter sequences. Unless specified, all bioinformatics steps above were done with default parameters.

### Ethics

Patient identifying data were not collected and privacy guaranteed. This report was started as part of the implementation of outbreak management strategies by the IPC team and followed the ORION checklist of items for outbreak reports (checklist provided in the Supplementary Materials) [9]. Furthermore, this research project was evaluated by the Ethics Committee Northwest and Central Switzerland (EKNZ) as not falling under the scope of the Human Research Act (Req-2024–0084 l). The planning conduct and reporting of this study was in line with the Declaration of Helsinki, as revised in 2013.

## Results

From November 2021 to June 2023, 152 *Klebsiella* spp. isolates were cultured from patient samples and 83 were genotyped using WGS (Table 2 and 3), along with six environmental isolates. This confirmed two outbreak waves, with multiple genotypically connected clusters during the second wave. 816 infants were screened for colonisation overall. 1117 blood cultures were collected over the surveillance period. Sixty (5.4%) tested positive, of which four (6.7%) grew *K. oxytoca*. All clinically relevant cases recovered after receiving antibiotic treatment.

**Table 2:** Characteristics of clinically relevant outbreak cases, with results of multilocus sequence typing (MLST) and K1 expression status

Sample code	Patient gestational age category at birth	Patient birth weight category	Length of stay at first positive sample (days)	Twin (yes)	Type of delivery	In/outborn	Diagnosis	Sample type	Pathogen	K1 [hOXY] phenotype	MLST	Antibiotic treatment (duration, days)	Outcome
NICUN002	Extremely preterm	ELBW	6		C-section	Inborn	Sepsis (late onset)	Blood	*K. oxytoca*	-	18	Amikacin + Amoxicillin-clavulanic acid iv (7 days)	Cured, alive
NICUN003	Very preterm	VLBW	5		Vaginal	Inborn	Sepsis (late onset)	Blood	*K. oxytoca*	-	18	Amikacin + Amoxicillin-clavulanic acid iv, shifted to Meropenem iv (14 days)	Cured, alive
NICUN013	Extremely preterm	ELBW	22	yes (twin A)	C-section	Inborn	Sepsis (late onset)	Blood	*K. oxytoca*	-	18	Amoxicillin-clavulanic acid (8 days) + Amikacin (3 days) Amikacin subsequently shifted to Meropenem iv (2 days)	Cured, alive
NICUN014	Extremely preterm	ELBW	26	yes (twin B)	C-section	Inborn	Clinically suspected invasive infection DD pneumonia	Tracheal secretion	*K. oxytoca* *(*concomitant *S. aureus, Acinetobacter junii,)*	K1	18	Amoxicillin-clavulanic acid + Amikacin iv (4 days), subsequently Piperacillin-tazobactam iv (7 days)	Cured, alive
NICUN037b	Extremely preterm	ELBW	10		Vaginal	Inborn	Clinically suspected invasive infection DD pneumonia	Tracheal secretion	*K. oxytoca* (later concomitant *S. aureus)*	-	199	Amoxicillin-clavulanic acid + Amikacin iv (14 days) shift to Ciprofloxacin po (6 days)	Cured, alive
NICUN038	Extremely preterm	ELBW	14		C-section	Inborn	Clinically suspected invasive infection DD pneumonia	Tracheal secretion	*K. oxytoca* (concomitant *S.aureus*)	K1	18	Amikacin + Amoxicillin-clavulanic acid iv (1 day), Shift to Meropenem + Vancomycin iv (7 days)	Cured, alive
NICUN069	Very preterm	VLBW	6		C-section	Inborn	Ventilator-associated Pneumonia	Tracheal secretion	*K. oxytoca* (concomitant *S.aureus*)	K1	18	Amikacin, Amoxicillin-clavulanic acid iv (4 days)	Cured, alive
NICUN081	Extremely preterm	ELBW	12	yes	C-section	Inborn	Ventilator-associated pneumonia	Tracheal secretion	*K. oxytoca* (concomitant MSSA)	-	199	Amoxicillin-clavulanic acid iv (5 days)	Cured, alive
NICUN083	Extremely preterm	ELBW	8		C-section	Inborn	Clinically suspected invasive infection	Tracheal secretion	*K. oxytoca*	-	199	Amikacin + Amoxicillin-clavulanic acid iv (4 days), Metronidazole iv (3 days) Shift to Vancomycin + Meropenem (5 days)	Cured, alive

Extremely preterm, born < 28 weeks’ gestational age, very preterm, born < 32 weeks’ gestational age, *VLBW* very low birth weight (< 1500 g), *ELBW* extremely low birth weight (< 1000 g), *C-section* caesarean section, *DD* differential diagnosis, *MLST* multilocus sequence type, *iv* intravenous

**Table 3:** Characteristics of asymptomatic outbreak cases, with results of multilocus sequence typing (MLST) and K1 expression status

Sample code	Patient gestational age category at birth	Patient birth weight category	Length of stay at first positive sample (days)	Twin (yes)	Type of delivery	In/outborn	Symptoms	Sample type	Pathogen	K1 [hOXY] phenotype	MLST	Antibiotic treatment (duration, days)	Outcome
NICUN004	Extremely preterm	ELBW	60		C-section	Inborn	-	Rectal swab	*K. oxytoca*	-	18	-	Alive
NICUN005	Very preterm	VLBW	28		C-section	Outborn	-	Rectal swab	*K. oxytoca*	-	18	-	Alive
NICUN006	Very preterm	VLBW	26		C-section	Inborn	-	Rectal swab	*K. oxytoca*	-	18	-	Alive
NICUN007	Moderately preterm	LBW	3		C-section	Inborn	-	Rectal swab	*K. oxytoca*	-	18	-	Alive
NICUN008	Very preterm	VLBW	14		C-section	Inborn	-	Rectal swab	*K. oxytoca*	-	18	-	Alive
NICUN009	Very preterm	VLBW	48		C-section	Inborn	-	Rectal swab	*K. oxytoca*	-	18	-	Alive
NICUN010	Very preterm	LBW	31		Vaginal	Inborn	-	Rectal swab	*K. oxytoca*	-	36	-	Alive
NICUN011	Extremely preterm	ELBW	107		C-section	Inborn	-	Rectal swab	*K. oxytoca*	-	37	-	Alive
NICUN015	Moderately preterm	VLBW	28		C-section	Inborn	-	Rectal swab	*K. oxytoca*	K1	18	-	Alive
NICUN016	Moderately preterm	LBW	3		C-section	Inborn	-	Rectal swab	*K. oxytoca*	K1	18	-	Alive
NICUN017	Moderately preterm	LBW	3		Vaginal	Inborn	-	Rectal swab	*K. oxytoca*	K1	18	-	Alive
NICUN018	Term	3000 g-4000 g	3		C-section	Inborn	-	Rectal swab	*K. oxytoca*	K1	18	-	Alive
NICUN019	Term	4000 g-5000 g	2		C-section	Inborn	-	Rectal swab	*K. oxytoca*	K1	18	-	Alive
NICUN020	Very preterm	VLBW	26	yes	C-section	Inborn	-	Rectal swab	*K. oxytoca*	K1	18	-	Alive
NICUN021	Very preterm	ELBW	26	yes	C-section	Inborn	-	Rectal swab	*K. oxytoca*	K1	18	-	Alive
NICUN022	Extremely preterm	VLBW	18	yes	C-section	Inborn	-	Rectal swab	*K. oxytoca*	K1	18	-	Alive
NICUN023	Extremely preterm	ELBW	18	yes	C-section	Inborn	-	Rectal swab	*K. oxytoca*	K1	18	-	Alive
NICUN024	Late preterm	LBW	27		C-section	Inborn	-	Rectal swab	*K. oxytoca*	K1	18	-	Alive
NICUN025	Late preterm	LBW	3		C-section	Inborn	-	Rectal swab	*K. oxytoca*	K1	18	-	Alive
NICUN027	Late preterm	LBW	1	yes	C-section	Inborn	-	Rectal swab	*K. oxytoca*	K1	18	-	Alive
NICUN028	Term	2500 g-3000 g	3	yes	C-section	Inborn	-	Rectal swab	*K. oxytoca*	K1	18	-	Alive
NICUN029	Term	LBW	3	yes	C-section	Inborn	-	Rectal swab	*K. oxytoca*	K1	18	-	Alive
NICUN030a	Very preterm	VLBW	3	yes	C-section	Inborn	-	Rectal swab	*K. oxytoca*	K1	18	-	Alive
									*K. grimontii*				
NICUN033	Extremely preterm	ELBW	3		C-section	Inborn	-	Rectal swab	*K. oxytoca*	-	199	-	Alive
NICUN034	Moderately preterm	2500 g-3000 g	3	yes	Vaginal	Inborn	-	Rectal swab	*K. oxytoca*	K1	18	-	Alive
NICUN035	Moderately preterm	2500 g-3000 g	3	yes	Vaginal	Inborn	-	Rectal swab	*K. oxytoca*	K1	18	-	Alive
NICUN036	Moderately preterm	LBW	13		C-section	Inborn	-	Rectal swab	*K. oxytoca*	-	199	-	Alive
NICUN039	Moderately preterm	LBW	3		C-section	Inborn	-	Rectal swab	*K. oxytoca*	-	199	-	Alive
NICUN043	Term	4040	4		Vaginal	Inborn	-	Rectal swab	*K. michiganensis*	-	194	-	Alive
NICUN045	Late preterm	LBW	3		Vaginal	Inborn	-	Rectal swab	*K. oxytoca*	K1	199	-	Alive
NICUN046	Term	3000 g-4000 g	3		Vaginal	Inborn	-	Rectal swab	*K. oxytoca*	K1	18	-	Alive
NICUN047	Late preterm	3000 g-4000 g	3		Vaginal	Inborn	-	Rectal swab	*K. oxytoca*	K1	18	-	Alive
NICUN048	Very preterm	VLBW	3		C-section	Inborn	-	Rectal swab	*K. oxytoca*	-	199	-	Alive
NICUN049	Late preterm	LBW	6		Vaginal	Outborn	-	Rectal swab	*K. michiganensis*	-	Insufficient results	-	Alive
NICUN050	Term	4000 g-5000 g	3		Vaginal	Inborn	-	Rectal swab	*K. oxytoca*	K1	18	-	Alive
NICUN051	Term	3000 g-4000 g	1		C-section	Inborn	-	Rectal swab	*K. oxytoca*	K1	18	-	Alive
NICUN052d	Extremely preterm	ELBW	41		C-section	Inborn	-	Rectal swab	*K. oxytoca*	K1	18	-	Alive
NICUN053	Term	3000 g-4000 g	3		Vaginal	Inborn	-	Rectal swab	*K. oxytoca*	-	199	-	Alive
NICUN054	Term	2500 g-3000 g	0	yes	Vaginal	Outborn	-	Rectal swab	*K. oxytoca*	K1	18	-	Alive
NICUN055	Term	LBW	3	yes	Vaginal	Outborn	-	Rectal swab	*K. oxytoca*	-	199	-	Alive
NICUN056	Moderately preterm	LBW	2		C-section	Inborn	-	Rectal swab	*K. oxytoca*	-	199	-	Alive
NICUN057	Extremely preterm	VLBW	8	yes	C-section	Inborn	-	Rectal swab	*K. oxytoca*	-	199	-	Alive
NICUN058	Extremely preterm	VLBW	8	yes	C-section	Inborn	-	Rectal swab	*K. michiganensis*	-	381	-	Alive
NICUN059	Late preterm	2500 g-3000 g	3		Vaginal	Outborn	-	Rectal swab	*K. oxytoca*	K1	18	-	Alive
NICUN060	Late preterm	LBW	1		C-section	Outborn	-	Skin Swab	*K. oxytoca*	K1	18	-	Alive
NICUN061	Term	3000 g-4000 g	1	yes	Vaginal	Outborn	-	Rectal swab	*K. oxytoca*	-	199	-	Alive
NICUN062	Term	3000 g-4000 g	3		Vaginal	Inborn	-	Rectal swab	*K. michiganensis*	-	381	-	Alive
NICUN063	Moderately preterm	LBW	2		C-section	Inborn	-	Anal swab	*K. oxytoca*	-	199	-	Alive
NICUN064	Moderately preterm	LBW	7		C-section	Inborn	-	Rectal swab	*K. michiganensis*	-	381	-	Alive
NICUN065	Extremely preterm	VLBW	7	yes	C-section	Inborn	-	Rectal swab	*K. oxytoca*	K1	18	-	Alive
NICUN066	Extremely preterm	VLBW	11	yes	C-section	Inborn	-	Rectal swab	*K. oxytoca*	K1	18	-	Alive
NICUN067c	Very preterm	LBW	3		C-section	Inborn	-	Rectal swab	*K. oxytoca*	-	199	-	Alive
NICUN068	Moderately preterm	LBW	3	yes	C-section	Inborn	-	Rectal swab	*K. oxytoca*	K1	18	-	Alive
NICUN070	Term	3000 g-4000 g	7		C-section	Outborn	-	Rectal swab	*K. oxytoca*	-	199	-	Alive
NICUN071	Term	3000 g-4000 g	4		Vaginal	Inborn	-	Rectal swab	*K. oxytoca*	K1	18	-	Alive
NICUN072	Very preterm	VLB	7	yes	C-section	Inborn	-	Rectal swab	*K. oxytoca*	-	199	-	Alive
NICUN073	Very preterm	ELBW	7	yes	C-section	Inborn	-	Rectal swab	*K. oxytoca*	-	199	-	Alive
NICUN074c	Very preterm	LBW	27		C-section	Inborn	-	Rectal swab	*K. oxytoca*	K1	18	-	Alive
NICUN075c										-	199		
NICUN076	Very preterm	VLBW	28		C-section	Inborn	-	Rectal swab	*K. oxytoca*	K1	18	-	Alive
NICUN077	Term	3000 g-4000 g	3		Vaginal	Outborn	-	Rectal swab	*K. oxytoca*	K1	18	-	Alive
NICUN078	Term	4000 g-5000 g	3		Vaginal	Inborn	-	Rectal swab	*K. michiganensis*	K1	52	-	Alive
NICUN079	Late preterm	LBW	7	yes	C-section	Inborn	-	Rectal swab	*K. oxytoca (concomita*nt *MSSA and Enterococcus* spp)	-	199	-	Alive
NICUN080	Late preterm	LBW	3	yes	C-section	Inborn	-	Rectal swab	*K. oxytoca*	-	199	-	Alive
NICUN082	Term	3000 g-4000 g	3		Vaginal	Inborn	-	Rectal swab	*K. oxytoca*	K1	18	-	Alive
NICUN084	Late preterm	LBW	3		Vaginal	Inborn	-	Rectal swab	*K. oxytoca*	-	199	-	Alive
NICUN085d	Extremely preterm	ELBW	41		C-section	Inborn	-	Urine	*K. oxytoca (*concomitant* E. coli)*	K1	18	-	Alive
NICUN086	Moderately preterm	LBW	3	yes	C-section	Inborn	-	Rectal swab	*K. oxytoca*	-	199	-	Alive
NICUN087	Moderately preterm	LBW	3	yes	C-section	Inborn	-	Rectal swab	*K. oxytoca*	-	199	-	Alive
NICUN088	Very preterm	VLBW	28	yes	C-section	Inborn	-	Rectal swab	*K. oxytoca*	-	199	-	Alive
NICUN089	Term	2500 g-3000 g	3		C-section	Inborn	-	Rectal swab	*K. oxytoca*	-	199	-	Alive
NICUN090	Very preterm	VLBW	3		C-section	Inborn	-	Rectal swab	*K. oxytoca*	-	199	-	Alive
NICUN091	Late preterm	LBW	3		C-section	Inborn	-	Rectal swab	*K. oxytoca*	K1	199	-	Alive
NICUN092	Term	3000 g-4000 g	4		C-section	Inborn	-	Rectal swab	*K. oxytoca*	-	151	-	Alive
									*K. michiganensis*				
NICUN093	Extremely preterm	ELBW	27		Vaginal	Inborn	-	Rectal swab	*K. oxytoca*	-	199	-	Alive

Extremely preterm, born < 28 weeks’ gestational age, very preterm, born < 32 weeks’ gestational age; moderately preterm, born between 32 and 34 weeks’ gestational age; late preterm, born between 34 and 37 week’s gestational age; term, born ≥ 37 weeks’ gestational age; *LBW* low birth weight (< 2500 g), *VLBW* very low birth weight (< 1500 g), *ELBW* extremely low birth weight (< 1000 g), *C-section* caesarean section, *MLST* multilocus sequence type, *iv* intravenous

### First outbreak wave

Chronologically, two *K. oxytoca* ST18 late-onset sepsis episodes with fully susceptible isolates occurred between November and December 2021, heralding the first wave of the outbreak lasting until February 2022. Following this alert to a potential outbreak, rectal swabs were taken from all patients and specifically tested for *K. oxytoca*. Of 10 potential outbreak cases identified between November 2021 and February 2022, two were identified as sequence type (ST) 36 and 37, while eight were wildtype ST18 confirmed by WGS to be part of an outbreak (two sepsis cases, six asymptomatic gut carriers).

### Outbreak containment measures

Because *K. oxytoca* has typically low virulence and a broad antimicrobial susceptibility, cases were neither cohorted nor isolated, in line with standard local IPC procedures. Targeted environmental sampling of water, sinks, syphons, bathtub, milk refrigerators, incubators and shared equipment was implemented during the outbreak window, revealing the bathtub, draining water via a permanently attached plastic hose, as a potential primary or intermediary source in February 2022 (infants were directly bathed; the bathtub was moved inside the rooms for bathing practices and placed in the NICU’s disposal unit whilst not in use. The bathtub would be filled with water from the sink in the disposal unit and emptied in the same sink). Upstream water sources were systematically considered in targeted, outbreak-specific environmental surveillance. Wildtype ST18 *K. oxytoca* was isolated from the bathtub syphon and water from hose (Table 4). The IPC team arranged for prompt replacement of the bathtub with a different model, without a drain or overflow.

**Table 4:** Positive environmental samples with results of multilocus sequence typing (MLST) and K1 expression status

Sample code	Source	First *Klebsiella* spp. positive sample date	Sample type	Pathogen	K1 [hOXY] phenotype	MLST
NICUE001	Bathtub	10.02.2022	Water from hose	*K. oxytoca*	-	18
NICUE002	Bathtub	10.02.2022	Water from hose	*K. oxytoca*	-	18
NICUE003	Bathtub	10.02.2022	Syphon	*K. oxytoca*	-	18
NICUE004	Washbasin—outside	11.10.2022	-	*K. oxytoca*	-	37
NICUE005	Milk refrigerators 1–3 (pooled)	11.10.2022	-	*K. oxytoca*	-	199
NICUE006	Washbasin—outside	20.12.2022	-	*K. michiganensis*	-	381

*MLST* multilocus sequence type

### Second outbreak wave

No further cases were notified to the IPC team until July 2022, when a set of twins was diagnosed with proven *K. oxytoca* ST18 sepsis (twin A) and colonisation with subsequent suspected pneumonia (twin B). The strain from twin B was identified as a K1-β-lactamase hyperproducer, showing a different resistance profile from the previously identified wildtype strain. WGS confirmed genotypic links of both strains to the first outbreak wave.

**Fig. 1:**
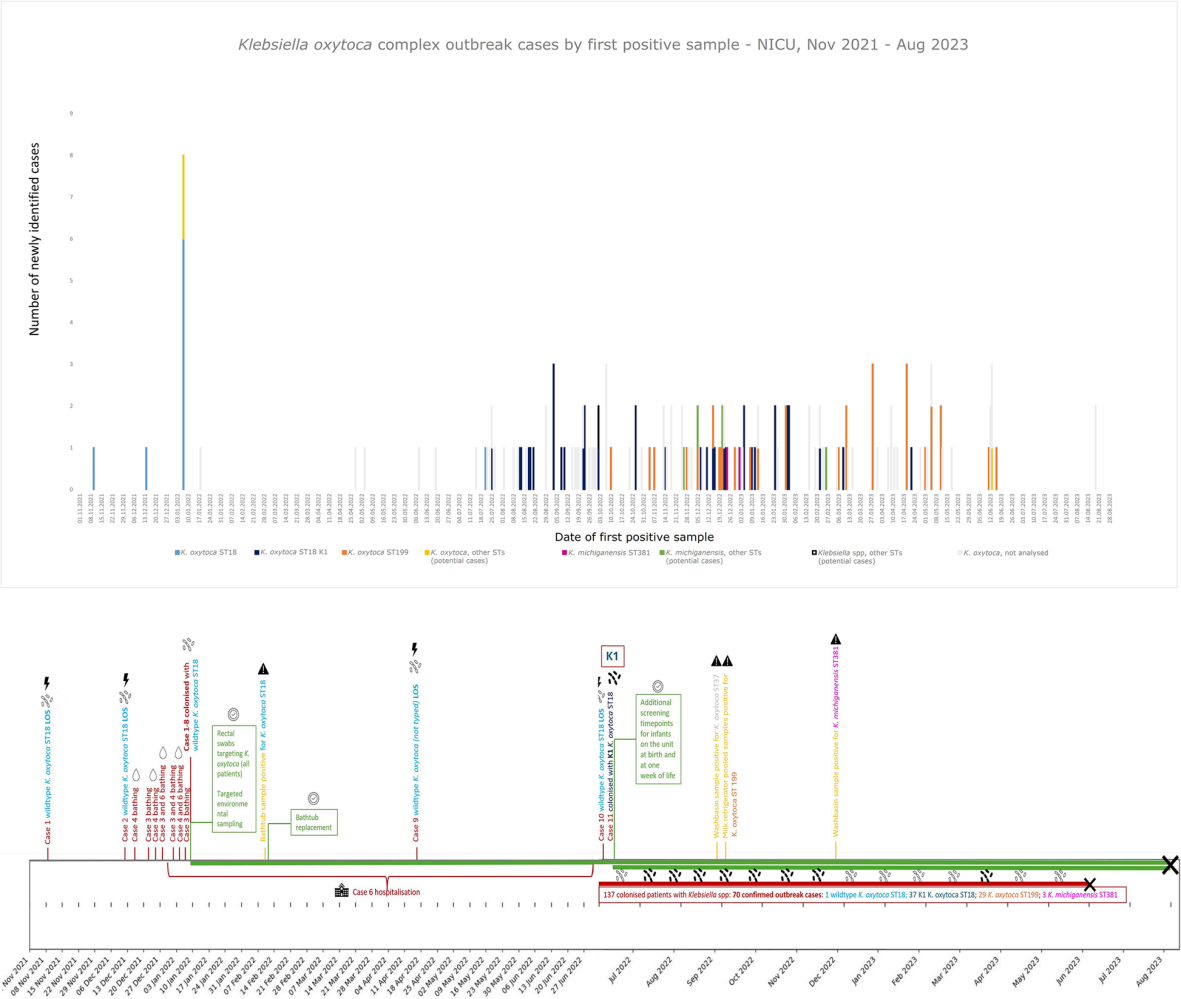
**A** Newly identified outbreak cases of *Klebsiella oxytoca* complex by first positive sample (November 2021 – August 2023); **B** Timeline of key outbreak events, environmental findings and IPC interventions. Wave 1: 10 cases (8 confirmed: *K. oxytoca* ST18; 2 potential: 1 K*. oxytoca* ST36, 1 K*. oxytoca* ST37). Wave 2: 73 cases (70 confirmed: 1 K*. oxytoca* ST18; 37 K*. oxytoca* ST18 K1; 29 K*. oxytoca* ST199; 3 K*. michiganensis* ST381; 3 potential: 1 K*. oxytoca* ST151; 1 K*. michiganensis* ST52; 1 K*. michiganensis* ST 194). Not typed: 69 isolates

Reviewing patient flow to and from the unit, one colonised infant of the first outbreak wave remained hospitalised until July 2022, likely promoting an outbreak resurgence despite elimination of an environmental source. A case of confirmed sepsis by a non-K1 *K. oxytoca* strain in April 2022 was retrospectively notified. This could not be sequenced but is likely linked to the outbreak. In addition, several infants had K1 phenotype Enterobacterales incidentally identified on routine screening samples in summer 2022. Additional screening timepoints for infants on the unit were instituted at birth and at one week of life. This delineated a second outbreak wave occurring from July 2022 to June 2023 (Fig. 1).

### Results of WGS typing

**Fig. 2:**
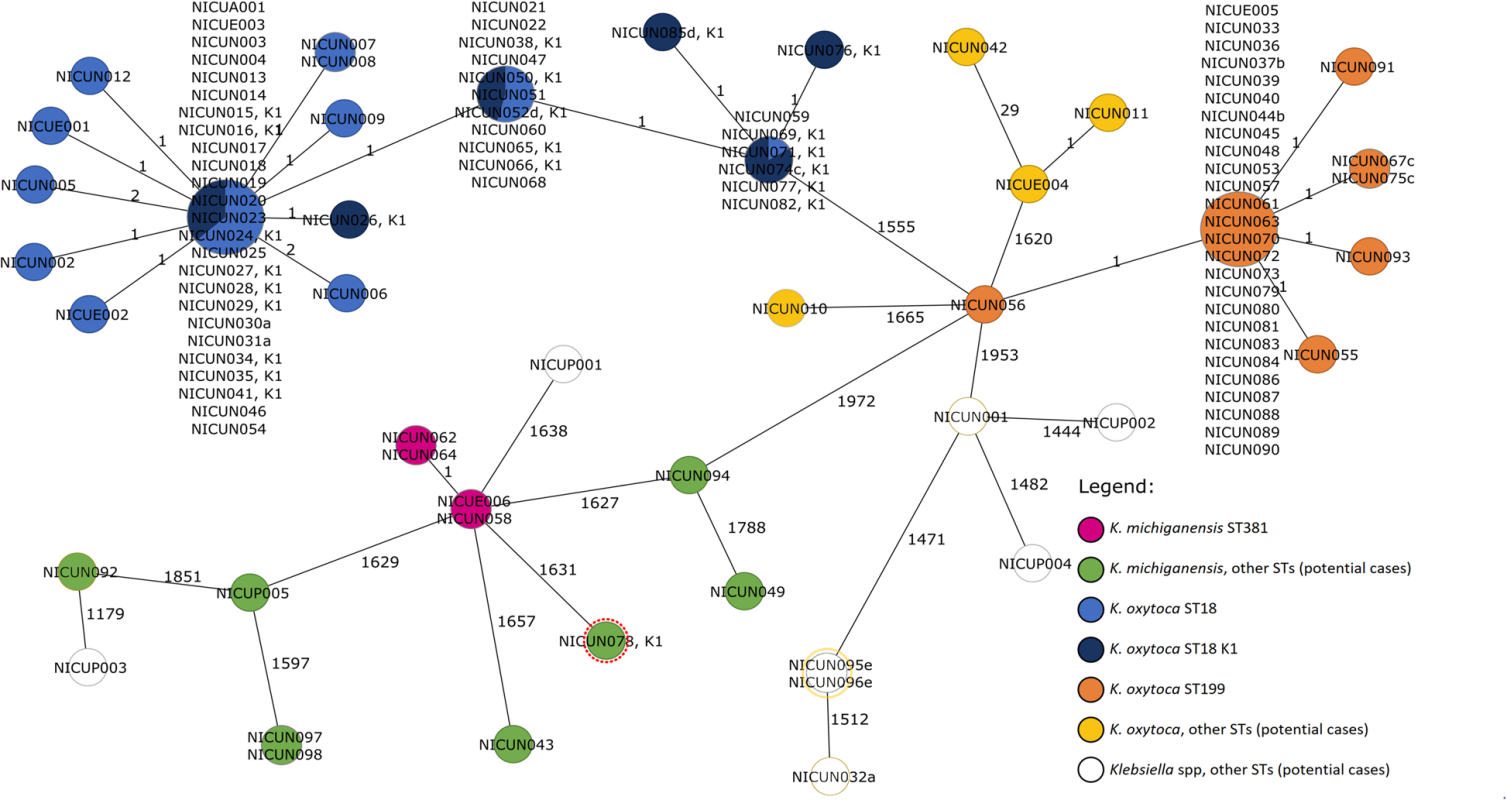
Minimum Spanning Tree of the Core Genome Multi Locus Sequencing Typing Analysis (cgMLST) – (samples: 2016–2024). The Minimum Spanning Tree of the cgMLST includes samples collected for surveillance and typed between 2016 and 2024, including those part of the reported outbreak. (NICUA, adult; NICUP, paediatric; NICUN, neonatal; NICUE, environmental; K1, associated with the K1 [hOXY] phenotype. The isolates are shown as circles. If two strains are identical, they collapse into one circle. The numbers on the lines connecting the different circles show the number of different alleles between two isolates (not to scales))

Of 137 patient samples growing *Klebsiella* spp since July 2022, 73 were typed, showing three concurrent clusters (Fig. 2). The main cluster was due to *K. oxytoca* ST18, with 38 confirmed cases, of which 37 were associated with the K1 [hOXY] phenotype. A second cluster was due to *K. oxytoca* ST199, with 29 related confirmed cases, mostly non-K1. ST199 was also found in pooled samples from the milk refrigerators (Table 4). Last, *K. michiganensis* was identified with a small cluster by ST381 (three colonised patients and one environmental sample from a washbasin). Of the patient samples that were not typed (samples not stored or unavailable, Supplementary Table S2), 33 were found to be K1-expressing. Overall, confirmed sepsis involving ST18 wildtype occurred in three very or extremely preterm, very/extremely low birth weight infants, who all recovered after receiving antibiotic treatment (Table 2). One further late-onset sepsis episode occurred in a very preterm infant, but the *K. oxytoca* isolate was not genotyped (Table S2).

The outbreak was declared contained by the end of June 2023 and the outbreak-specific surveillance was discontinued in August 2023. Support for optimal hand hygiene with electronic monitoring tools was only introduced in summer 2023 (overall average hand hygiene level was 35% for 105 NICU staff members between August and December 2023) [10]. A single point-prevalence survey was conducted for follow-up in February 2024, showing four infants with positive rectal swabs for *K. oxytoca* and *K. michiganensis*, with STs unrelated to the previous outbreaks (Table 5).

**Table 5:** Patients colonised with *Klebsiella* spp. at point-prevalence survey led in February 2024 (4/11 patients), with results of multilocus sequence typing (MLST) and K1 expression status

Sample code	Patient gestational age category at birth (weeks + days)	Patient birthweight category (g)	Length of stay at first positive sample (days)	Type of delivery	Inborn/outborn	Symptoms	Sample type	Pathogen	K1 [hOXY] phenotype	MLST	Antibiotic treatment (duration, days)	Outcome
NICUN094	Term	3000 g-4000 g	10	Vaginal	Outborn	**-**	Rectal swab	*K. michiganensis*	-	50	-	Alive
NICUN095e/96e	Term	2500 g-3000 g	5	C-section	Outborn	-	Rectal swab	*K. oxytoca*	-	186	-	Alive
								*K. grimontii*				
NICUN097	Moderately preterm	VLBW	1	C-section	Inborn	-	Rectal swab	*K. michiganensis*	-	409	-	Alive
NICUN098	Extremely preterm	VLBW	4	C-section	Inborn	-	Rectal swab	*K. michiganensis*	-	409	-	Alive

Extremely preterm, born < 28 weeks’ gestational age, moderately preterm, born between 32 and 34 weeks’ gestational age; term, born ≥ 37 weeks’ gestational age; *VLBW* very low birth weight (< 1500 g), *C-section* caesarean section, *MLST* multilocus sequence type

## Discussion

This report highlights some of the challenges of bacterial outbreaks in the NICU, especially those involving low-virulence ubiquitous pathogens. These become apparent when applying genome-based surveillance such as WGS.

Invasive infections were limited to higher-risk infants, with a 100% survival rate and no clearly attributable residual morbidity. In the context of multiple concurrent outbreaks being identified by WGS, the extensive use of measures such as cohorting, isolation or cot closures becomes less justifiable and feasible. Such findings align with previously published *K. oxytoca* complex neonatal outbreak reports (1984–2024), which mainly describe infant colonisation. Cases of neonatal infection were documented, especially bacteraemia in high-risk infants (mostly preterm or low birth weight), alongside colonisation, but affected few infants [3, 7, 11–28]. Case fatalities were also reported, though generally rare, and often following invasive infection [11, 13, 15, 19, 20, 23] or due to unrelated causes in fragile infants with multiple comorbidities [3, 26]. Cohorting and/or isolation of patients, and rarely cohorting of staff, were among the most frequently applied outbreak control measures according to published *K. oxytoca* outbreak reports (Table S1) [7, 11, 15, 18, 20, 22, 26, 27].

Several different genotyping and sequencing techniques have been described in the literature for microbial identification, outbreak characterisation and epidemiological surveillance. Techniques applied in *K. oxytoca* neonatal outbreaks, often with multiple techniques per study, were PCR-based methods [20–22, 24–26], DNA fingerprinting and comparative genotyping (Pulsed-field gel electrophoresis, PFGE; Restriction Fragment Length Polymorphism, RFLP or Small Fragment Restriction Endonuclease Analysis, SF-REA) [3, 7, 14, 15, 21, 23, 24, 27], next-generation sequencing (NGS), including WGS and 16S rRNA sequencing [15, 19, 21, 27, 28]. The latter have been increasingly applied over the last decade, notably WGS, which also plays a key role in the standardisation of prospective genomic surveillance for nosocomial pathogens and MDRO transmission [29].

Real-time WGS during outbreaks can enable faster identification of outbreak variants, differentiate between persistent environmental contamination versus patient-to-patient spread, thus informing tailored IPC interventions. This approach is resource-intensive, however lower-resource NICUs might adopt a phased approach (e.g. trigger-based WGS after case clusters). Combining real-time WGS of invasive isolates with trigger-based WGS of screening isolates would allow managing costs and maximising information gained. WGS revealed several observations of interest over the reported outbreak. First, it detected multiple concurrent outbreaks of previously unreported STs, especially during the second wave, proving effective in tracing transmission chains. *K. oxytoca* ST18 and ST199 and *K. michiganensis* ST381 were also identified in corresponding positive environmental samples. While WGS combined with epidemiological investigation and environmental sampling helped to identify an environmental source or potentiator, there was persistent onward infant-to-infant transmission linked to NICU-typical prolonged presence of colonised infants. The identification of environmental sources can be difficult and is variably described in published reports. Documented environmental sources include medical equipment and devices, such as blood gas analysers [11], disinfectant solutions [19, 28], humidifiers [21], water sources like sinks, drains/siphons and washing machines [7], feeding equipment or IV fluids, such as enteral nutrition tubes [20] and intravenous infusion bags [17]. However, not infrequently such sources are either purposely not investigated [3], not identified [15, 23, 26, 27]or not mentioned [12–14, 18, 22, 24, 25]. Outbreak investigations should consider upstream environmental testing. In our case, bathing practices and water exposure might have influenced transmission, leading to contamination and infant colonisation. No upstream contamination of water sources, however, was identified in this outbreak. Outbreaks often persist beyond interventions such as prompt removal of potentiators and additional measures, including enhanced environmental cleaning and disinfection, and even structural renovations. Likely multidirectional transfer-transmission between environmental sources and infants means that source identification and removal may not be sufficient to contain an outbreak. This highlights insufficient standard infection prevention and control measures to prevent transmission, often linked to understaffing and sub-optimal nurse-to-infant ratios*.*

Considering the STs identified in our study, *K. oxytoca* ST18 and ST199 isolates have been previously reported as being linked to clinical infections [30, 31]. *K. michiganensis* ST381 appears in both clinical and environmental samples, with plants representing a potential reservoir [30, 31]. Nevertheless, published studies specifically addressing neonatal outbreaks with *K. oxytoca* ST18, ST199 and *K. michiganensis* ST381 are lacking, with different STs being identified: *K. oxytoca* ST179 [27], ST201 [7], ST11, ST308, ST389, ST392 [3] and *K. michiganensis* ST50 [28].

Second, a set of twins presented a genotypically identical ST with a different susceptibility phenotype, wild-type vs. K1 (hyperproducer of OXY [hOXY-KoC], following point mutations in the consensus sequences of their promoters). This pertains to the emergence of resistance during an outbreak in a pathogen that is otherwise well-treatable. The K1 phenotype is typically susceptible to ceftazidime and cefepime, but resistant to cefuroxime, piperacillin and aztreonam. The distinction between the K1 and non-K1 phenotype is clearly associated with the published *bla*_*OXY*_ promoter sequences and *K. oxytoca* ST18 vs. non-ST18 [32]. Not all the collected samples were sequenced; however, it can be assumed that the non-typed K1 samples were likely ST18 and so part of the outbreak. The K1 phenotype was also described in a 2001 NICU outbreak in South Korea involving six infants, half of which presented with localised infection [21].

Third, differently from viral outbreaks easily contained by breaking the transmission chain, bacterial outbreaks have a greater risk of endemicity [33]. In fact, the duration of *K. oxytoca* outbreaks has been reported to range from 2 months to over a year [3, 7, 21–26].

If screening programmes are in place for MDRO/ESBL, it should be noted that K1 hyperproducers would be detected. This should then trigger an outbreak investigation if affecting more than one infant, even if the K1 hyperproducer is a “benign” *K. oxytoca*. We suggest the implementation of surveillance frameworks such as routine point prevalence surveys for colonisation and environmental sampling and targeted screening during outbreaks. Optimal and cost-efficient outbreak response, especially for low-virulence pathogens, must combine outbreak-specific investigations with maximisation of standard measures, such as optimal hand hygiene practices, including electronic monitoring tools. Hand hygiene with appropriate monitoring to boost compliance would significantly reduce multiple transfer-transmission events [10, 34, 35]. Various measures have been described for outbreak containment (Table S1), with reinforcement of hand hygiene through monitoring and training for optimal practice being one of the most frequently implemented actions [3, 7, 11, 12, 15, 18, 20, 22, 26, 28].

Limitations of this work are due to its retrospective design as an outbreak report. The actions described for outbreak surveillance and management reflect real-life practice and were not planned for the purpose of a study. Surveillance followed the criterion of infant time-since-admission on the ward (initially at 2–3 days of life and 4 weeks, additionally at birth and 1 week since the second wave), instead of absolute, pre-established regular screening timepoints, i.e. on a weekly basis. Moreover, WGS sequencing was implemented according to the local hospital practice, with limitations due to costs, and therefore it could not be performed for all collected samples. This could result in an incomplete picture of concurrent clusters, though all non-typed K1 samples were likely part of the main *K. oxytoca* ST18 cluster. Any lacking information would have been unlikely, however, to influence the management of the outbreak. Pathogens identified concomitantly to *Klebsiella* spp. on screening were not sequenced. Last, we did not perform a cost–benefit analysis comparing WGS-based surveillance and conventional approaches.

## Conclusion

To conclude, the use of WGS in the context of NICU outbreaks involving low-virulence bacteria enables precise mapping of transmission chains, revealing or refuting epidemiological links. It further enables tracking of evolution of resistance and its use can support identification and removal of potentiating environmental sources. Ongoing WGS surveillance of ubiquitous species may further uncover multiple concurrent outbreaks in the NICU, highlighting the added value of close collaboration between IPC and microbiology teams. Such collaborations enable the identification of tailored measures to address outbreak risks to patients and expected efficiency of IPC measures.

## Supplementary Information

Supplementary Material 1.

## Data Availability

The datasets generated and/or analysed during the current study are available in the text and Supplementary materials. The sequencing results have been uploaded on NCBI, under project number: PRJNA1198653 – Project title: “Whole-genome sequencing (WGS) of Klebsiella oxytoca clinical and environmental isolates from a Swiss neonatal unit.” Reviewer link: https://dataview.ncbi.nlm.nih.gov/object/PRJNA1198653?reviewer=8is7eai2mtd7v12jrchb7gl7v5.
