# Supplementary material for: Interpreting whole-genome sequencing data during neonatal Klebsiella oxytoca complex outbreak management
Source: Antimicrob Resist Infect Control. 2025 Jul 1;14:76. doi: 10.1186/s13756-025-01595-6 (PMC12219439; doi:10.1186/s13756-025-01595-6)
Supplement: Supplementary file 1 — Supplementary Material 1. [file 13756_2025_1595_MOESM1_ESM.docx]

# **Supplementary materials**

1. **Full search strategy**

**Plain search strategy**

(((((((neonat*) OR newborn) OR infant) OR babies)) AND ((((((infection) OR sepsis) OR septic) OR nosocomial infection) OR hospital-acquired infection) OR health-care associated infection)) AND (((outbreak) OR epidemic) OR cluster)) AND ((((((((center(s)) OR unit(s)) OR nursery) OR nurseries) OR hospital(s)) OR NICU) OR ward) OR neonatology) AND ((Klebsiella oxytoca) OR K. oxytoca)

**Ovid MEDLINE(R) ALL <1946 to January 22, 2025> : 17 results**

**1**  ((neonat* or newborn or infant or babies) and (infection or sepsis or septic or "nosocomial infection" or "hospital-acquired infection" or "health-care associated infection") and (outbreak or epidemic or cluster) and ((center* or unit* or nursery or nurseries or hospital* or NICU or ward or neonatology) and ("Klebsiella oxytoca" or "K. oxytoca"))).mp. [mp=title, book title, abstract, original title, name of substance word, subject heading word, floating sub-heading word, keyword heading word, organism supplementary concept word, protocol supplementary concept word, rare disease supplementary concept word, unique identifier, synonyms, population supplementary concept word, anatomy supplementary concept word] (17)

**Embase <1974 to 2025 January 22> : 39 results**

**1**  ((neonat* or newborn or infant or babies) and (infection or sepsis or septic or "nosocomial infection" or "hospital-acquired infection" or "health-care associated infection") and (outbreak or epidemic or cluster) and ((center* or unit* or nursery or nurseries or hospital* or NICU or ward or neonatology) and ("Klebsiella oxytoca" or "K. oxytoca"))).mp. [mp=title, abstract, heading word, drug trade name, original title, device manufacturer, drug manufacturer, device trade name, keyword heading word, floating subheading word, candidate term word] (39)

**Scopus Advanced: 257 results**

( ( ( ( ( neonat* ) OR newborn ) OR infant ) OR babies ) AND ( ( ( ( ( ( infection ) OR sepsis ) OR septic ) OR "nosocomial infection" ) OR "hospital-acquired infection" ) OR "health-care associated infection" ) AND ( ( ( outbreak ) OR epidemic ) OR cluster ) AND ( ( ( ( ( center ) OR unit ) OR nursery ) OR nurseries ) OR hospital ) OR nicu OR ward OR neonatology AND ( ( "Klebsiella oxytoca" ) OR ( "K. oxytoca" ) ) ) AND ( newborn ) AND ( LIMIT-TO ( DOCTYPE , "ar" ) OR LIMIT-TO ( DOCTYPE , "le" ) ) AND ( LIMIT-TO ( SUBJAREA , "MEDI" ) OR LIMIT-TO ( SUBJAREA , "NURS" ) OR LIMIT-TO ( SUBJAREA , "IMMU" ) ) AND ( LIMIT-TO ( EXACTKEYWORD , "Human" ) OR LIMIT-TO ( EXACTKEYWORD , "Klebsiella Oxytoca" ) )

1. **PRISMA flow diagram**


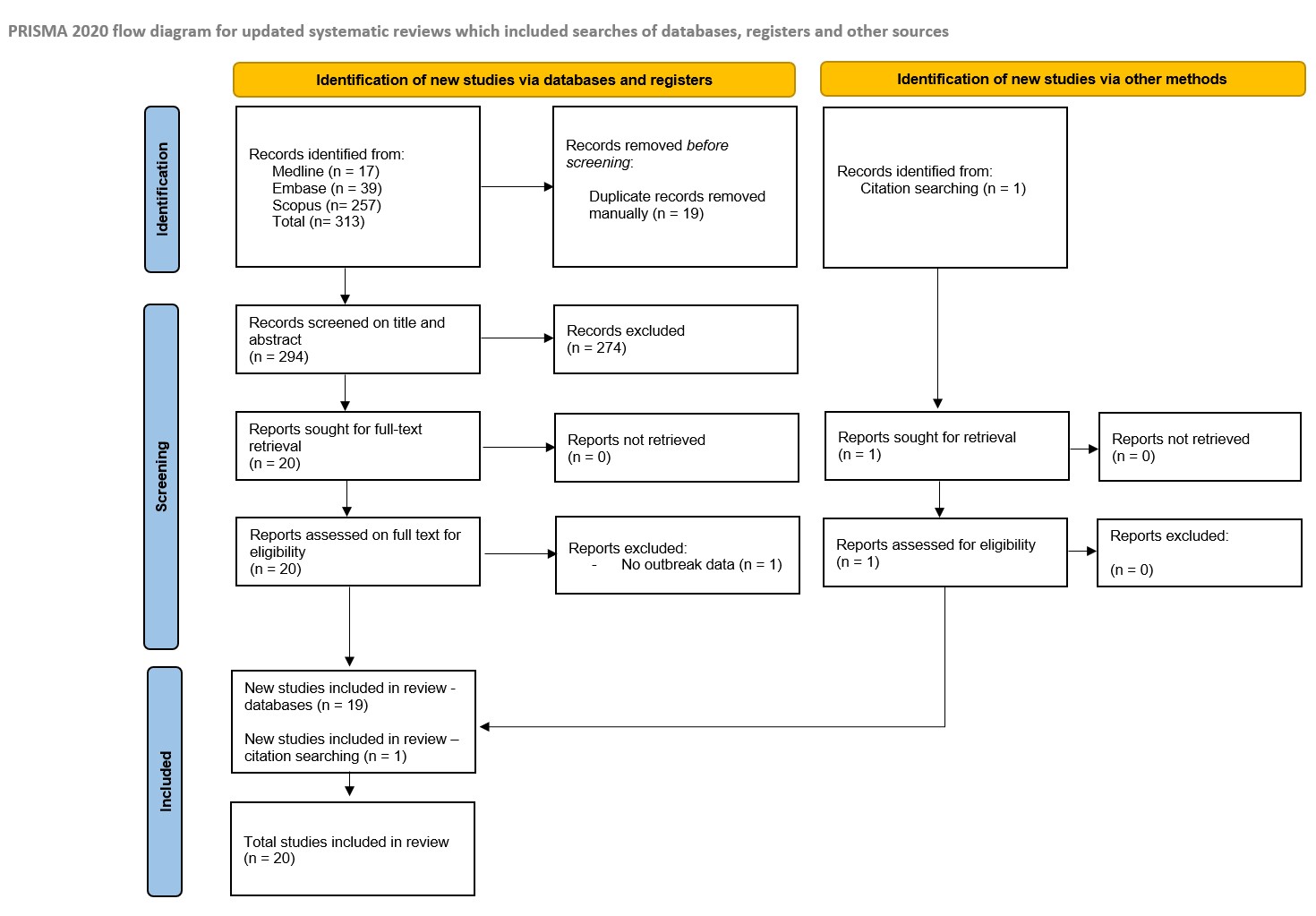


1. **Supplementary Tables**

**Table S1.** Summary of *K. oxytoca* NICU outbreaks included for review

| **Author (year)** | | | Morgan et al (1984)[11] | Aronsson et al. (1991)  [12] | Tullus et al. (1992)  [13] | Haertl et al. (1994)  [14] | Venezia et al. (1995)  [15] | Akindele et al. (1997)  [16] | Worku et al. (1997)[17] | Giramonti et al. (2000) [18] | Reiss et al. (2000) [19] | Berthelot et al. (2001)  [20] | Jeong et al. (2001) [21] | Ayan et al. (2003)  [22] | Kristóf et al. (2007) [23] | Grisold et al. (2010) [24] | Burak Selek (2014) [25] | Herruzo et al. (2017) [26] | Rønning et al. (2018) [27] | Schmithausen et al. (2019)  [3] | Chapman et al. (2020) [28] | Liébana-Rodríguez et al. (2024)  [2] |
| --- | --- | --- | --- | --- | --- | --- | --- | --- | --- | --- | --- | --- | --- | --- | --- | --- | --- | --- | --- | --- | --- | --- |
| **Country** | | | UK | Sweden | Sweden | Germany | USA | Nigeria | Ethiopia | USA | Germany | France | South Korea | Turkey | Hungary | Austria | Turkey | Spain | Norway | Germany | Australia | Spain |
| **Setting** | | | NICU | NICU | Neonatal special care wards | NICU | NICU | Neonatal units | Neonatal unit and Children’s ward | NICU | NICU | NICU | NICU | NICU | NICU | NICU | NICU | NICU | NICU | NICU | Special care nursery | NICU |
| **Population (infants, n.)** | | | 74 | 10 | 38 | 4 | 63 | 28 | 212 (isolates) | 15 | 24 | 18 | 6 | 10 | 21 | 6 | 8 | 20 | 22 | 13 | 10 | 21 |
| **Pathogen** | | | *K. oxytoca*  K55 | *K. oxytoca* | *K. oxytoca*  K55 | *K. oxytoca* | *K. oxytoca* | *Klebsiella* spp. (*K. oxytoca* n.1, 2.5%) | *K. oxytoca* | *K. oxytoca* | *K. oxytoca* | *K. oxytoca* | *K. oxytoca* | *K. oxytoca* | *K. oxytoca* | *K. oxytoca* | *K. oxytoca* | *K. oxytoca* | *K. oxytoca*  ST179 | *K. oxytoca*  ST 201 | *K. michiganensis*  ST50 | *K. oxytoca*  ST11; 308; 389; 392 |
| **Antibiotic resistance** | | | Gentamicin | Gentamicin | Cefuroxime | Gentamicin, cefazolin | ESBL (SHV-5  ) | Gentamicin  Netilmycin | Gentamicin | ESBL | / | / | K1 β-lactamase | ESBL (20%) | ESBL  (1: TEM-1, SHV-12;  2: SHV-5) | ESBL | ESBL | VIM carbapenemase | / | ESBL (CTX-M-15) | ESBL | / |
| **Infected (n., %)** | | | 12/74 (16.2%) | 7/10 (70%) | 38/38 (100%) | 1/4 (25%) | 3/63 (4.8%) | NR | 182/212 (86%) | 3/15 (20%) | 24/24 (100%) | 1/18 (5.6%) | 3/6 (50%) | 9/10 (90%) | 1: 9/9 (100%)  2:7/12 (58.3%) | NR | 8/8 (100%) | 4/20 (20%) | 5/22 (22.7%) | 0/13 (0%) | 0/10 (10%) | 6/21 (28.6%) |
| **Colonised (n., %)** | | | 62/74 (83.8%), stool | 3/10 (30%)  stool | 0/38 (0%) | 3/4 (75%) | 60/63 (95.2%) | NR | NR | 12/15 (80%) | 0/24 (0%) | 17/18 (94.4%) | 3/6 (50%) | 1/10 (10%) | 1: 0/9 (0%)  2: (5/12, 41.7%) | NR | 0/8 (0%) | 16/20 (80%) | 17/22 (77.3%) | 13/13 (100%)  Rectal and throat swabs | 10/10 (100%) | 15/21 (71.4%) |
| **Infection type** | | Bacteraemia/sepsis n. | 10/12 | 2/7 | 38/38 | 1/1 | 2/3 | NA | 182/212 | NR | 24/24 | 1/1 | / | 9/9 | NR | NR | / | / | 3/5 | / | / | / |
|  |  | Meningitis n. | 3/10 | / | / | / | / | NA | NR | NR | / | / | / | / | NR | NR | / | / | / | / | / | / |
|  |  | Pneumonia n. | / | 3/7 | / | / | / | NA | NR | NR | / | / | / | / | NR | NR | / | 3/4 | / | / | / | / |
|  |  | NEC n. | 2/12 | 1/7 | / | / | / | NA | NR | NR | / | / | / | / | NR | NR | / | / | 1/5 | / | / | / |
|  |  | UTI n. | / | / | / | / | 1/3 | NA | NR | NR | / | / | 1/3 | / | NR | NR | 8/8 | / | / | / | / | / |
|  |  | Wound n. | / | 1/7 | / | / | / | NA | NR | NR | / | / | / | / | NR | NR | / | / | / | / | / | / |
|  |  | Conjunctivitis n. | / | / | / | / | / | NA | NR | NR | / | / | 2/3 | / | NR | NR | / | 1/4 | 1/5 | / | / | 6/6 |
| **Clinical outcome** | | Dead (n., %) | 8/12 (66.7%) | NR | 5/38 (13.2%) | NR | 1/3 (33.3%) | NA | NR | NR | 2/24 (8.3%) | 1/18 (5.6%) | NR | NR | 3/29 (10.3%) | NR | NR | 1/20 (5%, other cause) | 0/22 (0%) | 0/13 (0%) | 0/10 (0%) | 1/21 (4.8%, other cause) |
|  |  | Cured/alive (n., %) | 4/12 (33.3%) | NR | 33/38 (86.8%) | NR | 2/3 (66.7%) | NA | NR | NR | 22/24 (91.7%) | 17/18 (94.4%) | NR | NR | 26/29 (89.7%9 | NR | NR | 19/20 (95%) | 22/22 (100%) | 13/13 (100%) | 10/10 (100%) | 20/21 (95.2%) |
| **Outbreak duration** | | | 29 weeks | 3 months | NR | NR | 30 weeks | NA | NR | NR | 2 years, 5 months | 8 months | 2 months | 2 months | 1: 5 months  2: 4 months | 2 months | 3 months | 15 months | 12 months | 14 months | 4 months | 4 months |
| **Probable outbreak source** | | | Blood gas analyser  Hand carriage | NR | NR | NR | Not identified | NA | Contaminated IV infusion bags | NR | Disinfectant solution | Enteral nutrition tube | Humidifiers | NR | Not identified | NR | NR | Not identified | Not identified | Washing machine  Siphons of staff sinks | Contaminated detergent | Not studied |
| **Genotyping/sequencing technique** | | | NA | NA | NA | SF-REA | RFLP by PFGE  Plasmid DNA sequencing | NA | NA | NA | Plasmid analysis and sequencing of 16S rRNA gene | AP-PCR | PCR and PFGE;  Nucleotide sequence analysis | AP-PCR | PFGE and plasmid isolation | PFGE and Rep-PCR | AP-PCR | DiversiLab PCR | PFGE and WGS | PFGE and MLST | WGS  Illumina NextSeq system | PFGE and sequencing |
| **IPC strategies** | Outbreak control team | |  |  | NR | NR |  | NR | NR |  |  | ● |  |  | NR | NR |  |  | ● |  |  | ● |
|  | Patients surveillance/screening | | ● | ● | NR | NR |  | NR | NR |  | ● | ● |  |  | NR | NR | ● | ● |  | ● | ● | ● |
|  | Mothers surveillance/screening | |  |  | NR | NR |  | NR | NR |  |  |  |  |  | NR | NR |  | ● |  | ● | ● |  |
|  | Staff surveillance/screening | |  | ● | NR | NR |  | NR | NR |  | ● |  |  |  | NR | NR | ● | ● |  | ● |  |  |
|  | Environmental surveillance/screening | | ● |  | NR | NR |  | NR | NR |  | ● |  |  |  | NR | NR | ● | ● |  | ● | ● |  |
|  | Contact/barrier precautions | | ● | ● | NR | NR | ● | NR | NR | ● |  | ● |  |  | NR | NR |  | ● |  |  | ● | ● |
|  | Cohorting/isolation (patients and/or/staff) | | ● |  | NR | NR | ● | NR | NR | ● |  | ● |  | ● | NR | NR |  | ● | ● | ● |  |  |
|  | Hand hygiene reinforcement (observations, training) | | ● | ● | NR | NR | ● | NR | NR | ● |  | ● |  | ● | NR | NR |  | ● |  | ● | ● | ● |
|  | Antimicrobial stewardship | |  | ● | NR | NR |  | NR | NR |  |  |  |  |  | NR | NR |  | ● |  |  |  |  |
|  | Potential source removal | |  |  | NR | NR |  | NR | NR |  | ● |  | ● |  | NR | NR |  |  | ● | ● | ● |  |
|  | Monitoring of newborn pathways | |  |  | NR | NR |  | NR | NR |  |  |  |  |  | NR | NR |  | ● |  | ● |  |  |
|  | Sterile water for bathing | |  |  | NR | NR |  | NR | NR |  |  |  |  |  | NR | NR |  |  |  | ● |  |  |
|  | Chlorhexidine 0.1-0.5% daily body washing | |  |  | NR | NR |  | NR | NR |  |  |  |  |  | NR | NR |  | ● |  |  |  |  |
|  | Disinfectant efficacy testing | |  |  | NR | NR |  | NR | NR |  |  |  |  |  | NR | NR |  | ● |  |  |  |  |
|  | Renovation/structural changes of ward rooms | |  |  | NR | NR |  | NR | NR |  |  |  |  |  | NR | NR |  |  |  | ● | ● | ● |
|  | Introduction of dispensers of disinfectant solutions | |  |  | NR | NR |  | NR | NR |  |  |  |  |  | NR | NR |  |  |  | ● |  | ● |
|  | Environmental cleaning/disinfecting procedures reinforcement | |  |  | NR | NR |  | NR | NR | ● |  |  | ● | ● | NR | NR |  |  | ● |  |  | ● |
|  | Monitoring of clinical procedures (i.e. management of central lines) | |  |  | NR | NR | ● | NR | NR |  |  |  |  |  | NR | NR |  |  | ● | ● | ● |  |
|  | Revision of breastmilk, formula milk and fortifiers handling | |  |  | NR | NR |  | NR | NR |  |  |  |  |  | NR | NR |  |  | ● |  |  |  |
|  | Pasteurisation of all human donor milk | |  |  | NR | NR |  | NR | NR |  |  |  |  |  | NR | NR |  |  | ● |  |  |  |
|  | Replacement of breast pumps and/or refrigerators | |  |  | NR | NR |  | NR | NR |  |  |  |  |  | NR | NR |  |  | ● |  |  |  |
| **Comments** | | | 63/164 (39%) infants on the ward carriers until discharge |  | IPC practices not discussed |  |  | Prospective review of antimicrobial susceptibility following outbreak occurred during severe water shortage in the city |  |  | Resistance against the disinfectant |  | IPC practices not further specified | High staff turnover; lack of equipment, shared cots | IPC practices not discussed |  | Isolates from patient samples differed from staff/environmental samples |  |  | Only infants who had worn clothes washed in the index washing machine were colonised | *K. oxytoca* identified by standard laboratory methods, identified as *K. michiganensis* at WGS |  |

Legend: UK, United Kingdom; USA. United States of America; IPC, infection prevention and control; NR, not reported; NA, not applicable; SF-REA, Small Fragment Restriction Endonuclease Analysis; NEC, necrotising enterocolitis; ESBL, extended-spectrum-β-lactamase producers ;UTI, urinary tract infection; AP-PCR, arbitrarily-prime polymerase chain reaction; ST, sequence type; RFLP, restriction fragment length polymorphism; PGE, pulsed-field gel electrophoresis; MLST, multilocus sequence typing; rep-PCR, repetitive-sequence-based PCR (DiversiLab); WGS, whole-genome sequencing

**Table S2.** Characteristics of cases not undergoing multilocus sequence typing (MLST), including K1 expression status.

| **Sample**  **code** | **Patient gestational age category at birth** | **Patient birth weight category** | **Length of stay at first positive sample (days)** | **Twin**  **(yes)** | **Type of delivery** | **In/outborn** | **Symptoms and/or diagnosis** | **Sample type** | **Pathogen** | **K1 [hOXY] phenotype** | **MLST** | **Antibiotic treatment (duration, days)** | **Outcome** |
| --- | --- | --- | --- | --- | --- | --- | --- | --- | --- | --- | --- | --- | --- |
| NICUU001 | Moderately preterm | LBW | 3 |  | Vaginal | Inborn | - | Rectal swab | *K. oxytoca* | - | Not analysed | - | Alive |
| NICUU003 | Very preterm | VLBW | 3 |  | C-section | Inborn | Sepsis (late onset) | Blood | *K. oxytoca* | - | Not analysed | Amikacin + Amoxicillin-clavulanic acid (6 days) + Metronidazole iv (4 days)    Amikacin + Amoxicillin-clavulanic acid iv  (4 days) | Cured, alive |
| NICUU004 | Extremely preterm | ELBW | 19 |  | C-section | Inborn | Conjunctivitis | Eye swab | *K. oxytoca* | - | Not analysed | Tobramycin, topic  (6 days) | Alive |
| NICUU005 | Extremely preterm | ELBW | 45 |  | C-section | Inborn | Clinically suspected invasive infection DD Neonatal pneumonia | Tracheal secretion | *K. oxytoca*  (concomitant *S. aureus)* | - | Not analysed | Amikacin iv (5 days) + Amoxicillin-clavulanic acid iv (8 days) | Cured, alive |
| NICUU006 | Very preterm | VLBW | 15 |  | C-section | Inborn | UTI | Urine | *K. oxytoca*  (on urine: concomitant *E. coli*) | - | Not analysed | Amikacin + Amoxicillin-clavulanic acid iv  (4 days) | Cured, alive |
| NICUU008 | Extremely preterm | ELBW | 27 |  | C-section | Inborn | - | Rectal swab | *K. oxytoca* | K1 | Not analysed | - | Alive |
| NICUU009 | Very preterm | VLBW | 4 |  | C-section | Inborn | - | Rectal/anal swab | *K. oxytoca* | K1 | Not analysed | - | Alive |
| NICUU010 | Extremely preterm | ELBW | 27 |  | C-section | Inborn | - | Rectal swab | *K. oxytoca* | K1 | Not analysed | - | Alive |
| NICUU011 | Moderately preterm | LBW | 3 |  | C-section | Inborn | - | Rectal/anal swab | *K. oxytoca* | K1 | Not analysed | - | Alive |
| NICUU012 | Moderately preterm | VLBW | 6 |  | C-section | Inborn | - | Ear swab | *K. oxytoca*  (concomitant *MSSA, S. epidermidis)* | - | Not analysed | - | Alive |
| NICUU013 | Late preterm | LBW | 3 |  | C-section | Inborn | - | Rectal swab | *K. oxytoca* | K1 | Not analysed | - | Alive |
| NICUU014 | Term | 3000g-4000g | 3 |  | Vaginal | Inborn | - | Rectal swab | *K. oxytoca* | K1 | Not analysed |  | Alive |
| NICUU015 | Term | 2500g-3000g | 20 |  | C-section | Inborn | - | Rectal swab | *K. oxytoca* | K1 | Not analysed | - | Alive |
| NICUU016 | Very preterm | VLBW | 26 |  | Vaginal | Inborn | - | Rectal swab | *K. oxytoca* | K1 | Not analysed | - | Alive |
| NICUU017 | Very preterm | ELBW | 4 |  | C-section | Inborn | - | Rectal swab | *K. oxytoca* | K1 | Not analysed | - | Alive |
| NICUU018 | Late preterm | 3000g-4000g | 3 |  | C.-section | Inborn | - | Rectal swab | *K. oxytoca* | K1 | Not analysed | - | Alive |
| NICUU019 | Term | 3000g-4000g | 2 |  | Vaginal | Outborn | - | Rectal swab | *K. oxytoca* | K1 | Not analysed | - | Alive |
| NICUU020 | Late preterm | LBW | 3 |  | Vaginal | Inborn | - | Rectal swab | *K. oxytoca* | K1 | Not analysed | - | Alive |
| NICUU021 | Very preterm | VLBW | 12 | yes | C-section | Inborn | - | Rectal swab | *K. oxytoca* | K1 | Not analysed | - | Alive |
| NICUU022 | Very preterm | LBW | 4 |  | C-section | Inborn | - | Rectal swab | *K. oxytoca* | K1 | Not analysed | - | Alive |
| NICUU023 | Moderately preterm | LBW | 9 |  | C-section | Inborn | - | Rectal swab | *K. oxytoca* | K1 | Not analysed | - | Alive |
| NICUU024 | Term | >5000g | 3 |  | C-section | Inborn | - | Anal swab | *K. oxytoca* | K1 | Not analysed | - | Alive |
| NICUU025 | Extremely preterm | ELBW | 58 |  | Vaginal | Outborn | - | Rectal swab | *K. oxytoca* | K1 | Not analysed | - | Alive |
| NICUU026 | Late preterm | LBW | 4 |  | Vaginal | Inborn | - | Rectal swab | *K. oxytoca* | K1 | Not analysed | - | Alive |
| NICUU027 | Very preterm | VLBW | 27 |  | C-section | Inborn | - | Rectal swab? | *K. oxytoca* | K1 | Not analysed | - | Alive |
| NICUU028 | Extremely preterm | ELBW | 3 |  | C-section | Inborn | - | Rectal swab | *K. oxytoca* | K1 | Not analysed | - | Alive |
| NICUU029 | Term | 3000g-4000g | 4 |  | C-section | Inborn | - | Rectal swab | *K. oxytoca* | - | Not analysed | - | Alive |
| NICUU030 | Term | 4000g-5000g | 3 |  | Vaginal | Outborn | - | Skin swab (groin) | *K. oxytoca* | K1 | Not analysed | - | Alive |
| NICUU031 | Term | 2500g-3000g | 3 |  | Vaginal | Outborn | - | Anal swab | *K. oxytoca* | K1 | Not analysed | - | Alive |
| NICUU032 | Extremely preterm | ELBW | 4 |  | C-section | Inborn | - | Rectal swab | *K. oxytoca* | K1 | Not analysed | - | Alive |
| NICUU033 | Extremely preterm | ELBW | 4 |  | C-section | Inborn | - | Rectal swab | *K. oxytoca* | - | Not analysed | - | Alive |
| NICUU034 | Extremely preterm | ELBW | 11 |  | C-section | Inborn | - | Rectal swab | *K. oxytoca* | - | Not analysed | - | Alive |
| NICUU035 | Term | 4000g-5000g | 3 |  | Vaginal | Inborn | - | Rectal swab | *K. oxytoca* | K1 | Not analysed | - | Alive |
| NICUU036 | Late preterm | LBW | 3 |  | C-section | Outborn | - | Rectal swab | *K. oxytoca* | - | Not analysed | - | Alive |
| NICUU037 | Extremely preterm | ELBW | 24 |  | C-section | Inborn | - | Rectal swab | *K. oxytoca* | K1 | Not analysed | - | Alive |
| NICUU038 | Moderately preterm | LBW | 11 |  | Vaginal | Inborn | - | Rectal swab | *K. oxytoca* | K1 | Not analysed | - | Alive |
| NICUU040 | Late preterm | LBW | 3 |  | C-section | Inborn | - | Rectal swab | *K. oxytoca* | K1 | Not analysed | - | Alive |
| NICUU041 | Term | LBW | 3 |  | C-section | Inborn | - | Rectal swab | *K. oxytoca* | K1 | Not analysed | - | Alive |
| NICUU042 | Late preterm | LBW | 3 |  | Vaginal | Inborn | - | Rectal swab | *K. oxytoca* | K1 | Not analysed | - | Alive |
| NICUU043 | Term | 3000g-4000g | 7 |  | C-section | Inborn | - | Rectal swab | *K. oxytoca* | - | Not analysed | - | Alive |
| NICUU044 | Term | 3000g-4000g | 7 |  | Vaginal | Inborn | - | Rectal swab | *K. oxytoca* | - | Not analysed | - | Alive |
| NICUU045 | Extremely preterm | VLBW | 8 |  | C-section | Inborn | - | Anal swab | *K. oxytoca* | - | Not analysed | - | Alive |
| NICUU046 | Very preterm | VLBW | 7 |  | C-section | Inborn | - | Rectal swab | *K. oxytoca* | K1 | Not analysed | - | Alive |
| NICUU047 | Term | 2500g-3000g | 0 |  | C-section | Outborn | - | Rectal swab | *K. oxytoca* | - | Not analysed | - | Alive |
| NICUU048 | Moderately preterm | LBW | 7 | yes | C-section | Inborn | - | Rectal swab | *K. oxytoca* | K1 | Not analysed | - | Alive |
| NICUU049 | Post-term | 4000g-5000g | 3 | yes | C-section | Inborn | - | Anal swab | *K. oxytoca* | - | Not analysed | - | Alive |
| NICUU050 | Moderately preterm | LBW | 7 |  | Vaginal | Inborn | - | Rectal swab | *K. oxytoca* | K1 | Not analysed | - | Alive |
| NICUU051 | Term | 4000g-5000g | 2 |  | Vaginal | Outborn | - | Rectal swab | *K. oxytoca* | K1 | Not analysed | - | Alive |
| NICUU052 | Extremely preterm | ELBW | 9 |  | C-section | Inborn | - | Nasal secretion | *K. oxytoca* | - | Not analysed | - | Alive |
| NICUU053 | Very preterm | VLBW | 3 |  | C-section | Inborn | - | Rectal swab | *K. oxytoca* | - | Not analysed | - | Alive |
| NICUU054 | Extremely preterm | ELBW | 2 | yes | C-section | Inborn | - | Rectal swab | *K. oxytoca* | - | Not analysed | - | Alive |
| NICUU055 | Term | 3000g-4000g | 6 |  | Vaginal | Outborn | - | Rectal swab | *K. oxytoca* | - | Not analysed | - | Alive |
| NICUU056 | Moderately preterm | LBW | 7 |  | C-section | Inborn | - | Rectal swab | *K. oxytoca* | - | Not analysed | - | Alive |
| NICUU057 | Term | 4000g-5000g | 3 |  | Vaginal | Inborn | - | Anal swab | *K. oxytoca* | - | Not analysed | - | Alive |
| NICUU058 | Very preterm | ELBW | 27 |  | C-section | Inborn | - | Rectal swab | *K. oxytoca* | - | Not analysed | - | Alive |
| NICUU059 | Very preterm | LBW | 3 | yes | C-section | Inborn | - | Rectal swab | *K. oxytoca* | - | Not analysed | - | Alive |
| NICUU060 | Very preterm | ELBW | 3 |  | C-section | Inborn | - | Rectal swab | *K. oxytoca* | - | Not analysed | - | Alive |
| NICUU061 | Term | LBW | 3 |  | C-section | Inborn | - | Rectal swab | *K. oxytoca* | - | Not analysed | - | Alive |
| NICUU062 | Extremely preterm | ELBW | 27 |  | C-section | Inborn | - | Rectal swab | *K. oxytoca* | - | Not analysed | - | Alive |
| NICUU063 | Very preterm | LBW | 26 |  | C-section | Inborn | - | Rectal swab | *K. oxytoca* | - | Not analysed | - | Alive |
| NICUU065 | Extremely preterm | ELBW | 3 |  | C-section | Inborn | - | Rectal swab | *K. oxytoca (R ampi and Ceftriaxone)* | - | Not analysed | - | Alive |
| NICUU066 | Extremely preterm | ELBW | 4 |  | Vaginal | Inborn | - | Rectal swab | *K. oxytoca* | - | Not analysed | - | Alive |
| NICUU067 | Extremely preterm | ELBW | 8 |  | C-section | Inborn | Ventilator-associated Pneumonia | Tracheal secretion | *K. oxytoca* | - | Not analysed | Amikacin + Amoxicillin-clavulanic acid iv  (5 days) | Cured, alive |
| NICUU068 | Moderately preterm | LBW | 3 |  | C-section | Inborn | - | Rectal swab | *K. oxytoca* | - | Not analysed | - | Alive |
| NICUU069 | Moderately preterm | 3000g-4000g | 3 |  | Vaginal | Inborn | - | Rectal swab | *K. oxytoca* | - | Not analysed | - | Alive |
| NICUU070 | Late preterm | LBW | 3 |  | Vaginal | Inborn | - | Rectal swab | *K. oxytoca* | - | Not analysed | - | Alive |
| NICUU071 | Very preterm | LBW | 27 |  | Vaginal | Inborn | - | Rectal swab | *K. oxytoca* | - | Not analysed | - | Alive |
| NICUU072 | Moderately preterm | LBW | 3 |  | C-section | Inborn | - | Rectal swab | *K. oxytoca* | - | Not analysed | - | Alive |

**Legend:** extremely preterm, born <28 weeks’ gestational age, very preterm, born <32 weeks’ gestational age; moderately preterm, born between 32 and 34 weeks’ gestational age; late preterm, born between 34 and 37 week’s gestational age; term, born ≥ 37 weeks’ gestational age; LBW, low birth weight (<2500g); VLBW, very low birth weight (<1500g); ELBW, extremely low birth weight (<1000g); C-section, cesarean section; DD= differential diagnosis, UTI= urinary tract infection; MLST= multilocus sequence type; iv, intravenous.

This supplementary material contains supporting information alongside the article entitled “Interpreting whole-genome sequencing data during neonatal *Klebsiella oxytoca* outbreak management”, on behalf of the authors, who remain responsible for the accuracy and appropriateness of the content.

1. ORION Checklist of items to include when reporting an outbreak or intervention study of a nosocomial organism

DONE

|  | **Item Number** | **Descriptor**  - INAPPROPRIATE / NOT APPLICABLE |
| --- | --- | --- |
| **Title & Abstract** | 1 | Description of paper as outbreak report or intervention study.  Design of intervention study (eg Randomised Controlled Trial , Cluster Randomised Controlled Trial, Interrupted Time Series, Cohort study etc). Brief description of intervention and main outcomes. - DONE  group.  ____ |
| **Introduction**  Background | 2 | Scientific and/or local clinical background and rationale.  Description of organism as epidemic, endemic or epidemic becoming endemic. - DONE |
| Type of paper | 3 | Description of paper as Intervention study or an Outbreak Report. If an outbreak report, report the number of outbreaks. - DONE |
| Dates | 4 | Start and finish dates of the study or report. - DONE  - DONE |
| Objectives | 5 | Objectives for outbreak reports. Hypotheses for intervention studies - DONE |
| **Methods**  Design | 6 | Study design. Use of EPOC classification recommended (RCT or CRCT, CBA, or ITS)  - DONE  Whether study was retrospective, prospective or ambidirectional - DONE  Whether decision to report or intervene was prompted by any outcome data.  NOT APPLICABLE  NOT APPLICABLE  Whether study was formally implemented with predefined protocol and endpoints. |
| Participants | 7 | Number of patients admitted in study or outbreak. Summaries of distributions of age and lengths of stays. If possible, proportion admitted from other wards, hospitals, nursing homes or from abroad. Where relevant, potential risk factors for acquiring the organism. Eligibility criteria for study. Case definitions for outbreak report. -DONE |
| Setting | 8 | Description of the unit, ward or hospital and, if a hospital, the units included.  Number of beds, the presence and staffing levels of an infection control team. - DONE |
| Interventions | 9 | Definition of phases by major change in specific infection control practice (with start and stop dates). A summary table is strongly recommended with precise details of interventions, how and when administered in each phase. - DONE  - |
| Culturing & Typing | 10 | Details of culture media, use of selective antibiotics and local and /or reference typing. Where relevant, details of environmental sampling. - DONE |
| Infection-related outcomes | 11 | Clearly defined primary and secondary outcomes (eg incidence of infection, colonisation , bacteraemia) at regular time intervals (eg daily, weekly, monthly) rather than as totals for each phase, with at least three data points per phase and, for many two phase studies, 12 or more monthly data points per phase. Denominators (eg numbers admissions or discharges, patient bed days). If possible, prevalence of organism and incidence of colonisation on admission at same time intervals. Criteria for infection, colonisation on admission and directly attributable mortality.  For short studies or outbreak reports, use of charts with duration patient stay & dates organism detected may be useful (see text) - DONE  ers  - NOT APPLICABLE |
| Economic outcomes | 12 | If a formal economic study done, definition of outcomes to be reported, description of resources used in interventions, with costs broken down to basic units, stating important assumptions.- NOT APPLICABLE |
| Potential Threats to internal validity | 13 | Which potential confounders were considered, recorded or adjusted for (eg: changes in length of stay, case mix, bed occupancy, staffing levels, hand-hygiene compliance, antibiotic use, strain type, processing of isolates, seasonality).  Description of measures to avoid bias including blinding & standardisation of outcome assessment & provision of care. - DONE  - NOT APPLICABLE |
| Sample size | 14 | Details of power calculations, where appropriate – NOT APPLICABLE |
| Statistical methods | 15 | Description of statistical methods to compare groups or phases. Methods for any subgroup or adjusted analyses, distinguishing between planned and unplanned (exploratory) analysis. Unless outcomes are independent, statistical approaches able to account for dependencies in the outcome data should be used, adjusting, where necessary, for potential confounders  For outbreak reports statistical analysis may be inappropriate. – NOT APPLICABLE |
| **Results**  Recruitment | 16 | For relevant designs the dates defining periods of recruitment and follow-up. A flow diagram is recommended to describe participant flow in each stage of study. - NOT APPLICABLE |
| Outcomes & estimation | 17 | For the main outcomes, the estimated effect size and its precision (usually using confidence intervals). A graphical summary of the outcome data is often appropriate for dependent data (such as most time series). – NOT APPLICABLE |
| Ancillary analyses | 18 | Any subgroup analyses should be reported and it should be stated whether or not it was planned (specified in the protocol) and possible confounders adjusted for –   - NOT APPLICABLE |
| Adverse events | 19 | Pre-specified categories of adverse events and occurrences of these in each intervention group. This might include drug side effects, crude or disease specific mortality in antibiotic policy studies or opportunity costs in isolation studies. - DONE |
| **Discussion**  Interpretation | 20 | For intervention studies an assessment of evidence for/against hypotheses, accounting for potential threats to validity of inference including regression to mean effects and reporting bias. – NOT APPLICABLE  For outbreak reports, consider clinical significance of observations and hypotheses generated to explain them. -DONE |
| Generalisability | 21 | External validity of the findings of the intervention study i.e. to what degree can results be expected to generalise to different target populations or settings. - DONE |
| Overall evidence | 22 | General interpretation of results in context of current evidence. - DONE |
| Abbreviations: RCT: randomised controlled trial; CRCT: Cluster Randomised Controlled Trial; CBA: controlled before and after study; ITS: interrupted time series | | |
